# Supplementary material for: Modifiable Maternal Factors and Their Relationship to Postpartum Depression
Source: Int J Environ Res Public Health. 2022 Sep 29;19(19):12393. doi: 10.3390/ijerph191912393 (PMC9564437; doi:10.3390/ijerph191912393)
Supplement: Supplementary file 1 [file ijerph-19-12393-s001.zip › ijerph-1917497-supplementary.pdf]

Supplementary Materials:

The results from BRUMS and EPDS showed a strong correlation between PPD and every mood category at six months postpartum (and in the appropriate direction), suggesting the data collection instruments are valid. All but four were correlated at 12 months, but this is likely due to the reduced sample size at 12 months.

Table S1. Relationships between PPD (assessed by the EPDS) and the constructs of the BRUMS .

**6 months**										
**Anger**	**Tension**	**Depression**	**Vigor**	**Fatigue**	**Confusion**	**Happy**	**Calmness**	**PBW**	**PD**	FAT
0.76	0.744	0.782	-0.553	0.700	0.629	-0.625	-0.731	-0.474	0.407	0.709
<0.001	<0.001	<0.001	0.008	<0.001	0.002	0.002	<0.001	0.022	0.054	<0.001
12 months										
Anger	Tension	Depression	Vigor	Fatigue	Confusion	Happy	Calmness	PBW	PD	FAT
0.308	0.364	0.637**	-0.388	0.680**	0.601*	-0.463	-0.624**	-.0702**	0.763**	0.719**
0.245	0.166	.008	0.138	0.004	0.014	0.071	0.010	.0002	<.001	0.001

------- *PWB = Positive Well-Being, PD = physiological distress, FAT = fatigue*; *p<0.05; **P<0.001
